# Organic Food Consumption and Risk of Obesity: A Systematic Review and Meta-Analysis

**DOI:** 10.3390/healthcare10020231

**Published:** 2022-01-26

**Authors:** Akshaya Srikanth Bhagavathula, Kota Vidyasagar, Jagdish Khubchandani

**Affiliations:** 1 Department of Social and Clinical Pharmacy, Faculty of Pharmacy at Hradec Kralove, Charles University, 50003 Hradec Kralove, Czech Republic; akshaypharmd@gmail.com; 2 Department of Pharmaceutical Sciences, University College of Pharmaceutical Sciences, Hanamkonda 506009, India; vidyasagarkota2@gmail.com; 3 Department of Public Health Sciences, College of Health, Education, and Social Transformation, New Mexico State University, Las Cruces, NM 88003, USA

**Keywords:** food, diet, nutrition, lifestyle, obesity, overweight, behavior

## Abstract

Recent epidemiological studies have explored the association between organic food consumption and the risk of obesity, but the results remain controversial. A systematic review and meta-analysis were conducted to determine the association between organic food consumption and the risk of obesity. Rigorous methods for a comprehensive search were employed to search for literature in PubMed/MEDLINE, Web of Science, and Embase for relevant articles published until 30 November 2021. Pooled odds ratio (OR) with 95% confidence intervals (Cis) were calculated using a DerSimonian and Laird random-effects model to understand the risk of obesity based on exposure to organic food. Four studies, comprising 104,488 healthy subjects and 39,425 adults who consumed organic food, reported 1625 incident cases of obesity. Compared with the unexposed group, organic food consumption was associated with a lower probability of obesity (OR: 0.89, 95% CI: 0.80–0.97, *p* < 0.001). Subgroup analysis showed that this association was higher in the cohort (OR: 0.78, 95% CI: 0.63–0.92) than cross-sectional studies (OR: 0.95, 95% CI: 0.91–1.00), respectively. Overall, organic food consumption had a modest reduction (11%) in the risk of obesity and can be an appropriate strategy to prevent obesity.

## 1. Introduction

In the 21st century, obesity has emerged as one of the most serious public health concerns worldwide [1,2]. Recent estimates have suggested that more than 500 million adults are obese worldwide, and nearly four million individuals die annually due to a high body mass index (BMI) [1]. As a result, cardiovascular diseases and diabetes have also become the leading causes of mortality in general and in association with high BMI [1,2,3]. In addition to premature mortality, obesity is also associated with various comorbidities and chronic diseases ranging from physical to mental health issues and considerable loss of quality of life and disabilities [1,3,4,5]. Given the high burden of obesity and associated morbidity and mortality, several studies and interventions have been conducted to address the rising epidemic of obesity. Most of these interventions and recommendations are designed to reduce calorie consumption, increase fruit and vegetable consumption, and improve physical activity and exercise [3,4,5,6].

Given the overwhelming strategies and interventions to improve health and manage body weight, there has been much interest recently in new dietary habits, supplements, and alternate diets. A prominent entity in this regard is “organic food.” Touted as one of the biggest consumer trends over the past few decades, the organic movement has gained particular popularity in western countries [7,8,9]. For example, in a 2016 survey, most Americans (68%) reported that they had bought organic food at least once in the preceding month. In addition, 40% of Americans reported that most or some of the foods they ate were organic [8]. Similarly, the global sales of organic products have increased sharply in the 21st century. According to one report, in 2019, sales of organic food amounted to USD 106 billion, up from USD 18 billion in 2000 [9]. It is postulated that traditional foods may be exposed to chemicals used to produce food that increase the risk of health problems. These chemicals (e.g., pesticides) promote toxicity risks towards human health but can cause many subtle metabolic and endocrine changes that collectively pose long-term health risks, including obesity and metabolic disorders. In contrast, organic foods are predominantly cultivated, in practice, free from chemical exposure to the harvest causing a reduction in the risk of health problems [7,10,11,12]. Despite the growing popularity and the proliferation of media reports and articles on the benefits of organic food, evidence-based research on the benefits and effects of organic food has recently started emerging in the scientific literature. One of the earliest and most rigorous reviews of organic food found that consumption of organic foods may reduce exposure to pesticide residues and antibiotic-resistant bacteria [10]. However, the review lacked strong evidence that organic foods are significantly more nutritious than conventional foods. In contrast, more recent reviews suggest that organic foods may be healthier and of higher nutritional value [11,12]. Despite the various findings, all these studies unanimously suggest the need for better assessment and evidence synthesis on the benefits of organic foods, such as the influence on body mass index [10,11,12]. The purpose of this review was to investigate the association between the consumption of organic food and the risk of obesity.

## 2. Materials and Methods

A systematic review and meta-analysis were conducted following the Meta-analysis of Observational Studies in Epidemiology (MOOSE) guidelines [13].

### 2.1. Literature Search Strategy

A literature search was performed to identify relevant available articles published in the English language from PubMed/MEDLINE, Web of Science, and Embase up to 30 November 2021, using search terms: “organic food” (or “organic products” or “organic diet” or “ organic diet”) and “obesity” (or “obese” or “obesities” or “excess body weight”). Reference lists of the included articles were also checked to identify further relevant studies.

### 2.2. Inclusion and Exclusion Criteria

We included cross-sectional and cohort studies investigating the association between organic food consumption and the incidence of obesity in healthy subjects. Briefly, the inclusion criteria were as follows: (1) a published observational study; (2) organic food or organic products as the exposure of interest; (3) obesity as the outcome of interest; (4) odds ratios (ORs) or relative risks (RRs) or hazard ratios (HRs) with 95% confidence intervals (CI) were provided; (5) the most recent study was selected if data from the same population had been published more than once. Studies that did not provide sufficient information on the exposure and outcome of interest were not considered. Studies conducted on special populations (children, pregnant women, older) and diseased populations were excluded. Non-human studies, in vitro research, review articles, editorials, letters to editors, case reports or case series, and letters without sufficient data were also excluded from this study.

### 2.3. Data Extraction

Two reviewers (ASB and KVS) independently reviewed the articles and extracted the relevant data. The following information was extracted: (1) the first author’s name; (2) year of publication; (3) study region; (4) study design; (4) study population; (5) sample size; (6) age range or mean age; (7) definition of obesity; (8) duration of follow-up and the cases of obesity; (9) organic food consumption; (10) variables adjusted for each study and (11) OR (we presented all results as OR for simplicity) with 95% CI. Moreover, OR of the highest threshold of organic food consumption, adjusted for confounders, was extracted. The quality assessment of observational studies was conducted using the Newcastle-Ottawa Quality Assessment Scale (NOS) [14]. Studies with a NOS scale score of ≥7 were considered high-quality studies. Disagreement at each stage was resolved by consensus and involving a third author (JK) if required.

## 3. Statistical Analysis

The data analysis was performed using Stata version 16 (StataCorp, College Station, TX, USA). Risk estimates comprising higher quartiles from the most adjusted models were used for analysis. To investigate the strength of the association between organic food consumption and the risk of obesity, ORs were naturally log-transformed and pooled using DerSimonian and Laird random-effects models [15]. When studies did not provide standard error (SE) or 95% confidence intervals (CIs) of the mean for calculating the effect size directly, it was estimated from published data using the methods described by Greenland [16]. *I*^2^ was used to assess the heterogeneity between studies (*I*^2^ values of 0%, 25%, 50%, and 75% represent no, low, moderate, and high heterogeneity, respectively), and a *p* < 0.10 was considered evidence of substantial heterogeneity [17]. Subgroup analysis was performed to explore the sources of heterogeneity. The effect of each study in the overall estimates was evaluated by sensitivity analysis. Statistical methods cannot detect publication bias when the number of available published studies was small (<10), thus publication bias was not assessed [17]. All reported probabilities (*p*-values) were two-tailed, and a *p*-value of <0.05 was considered statistically significant.

## 4. Results

### 4.1. Literature Search and Study Characteristics

**Figure 1:**
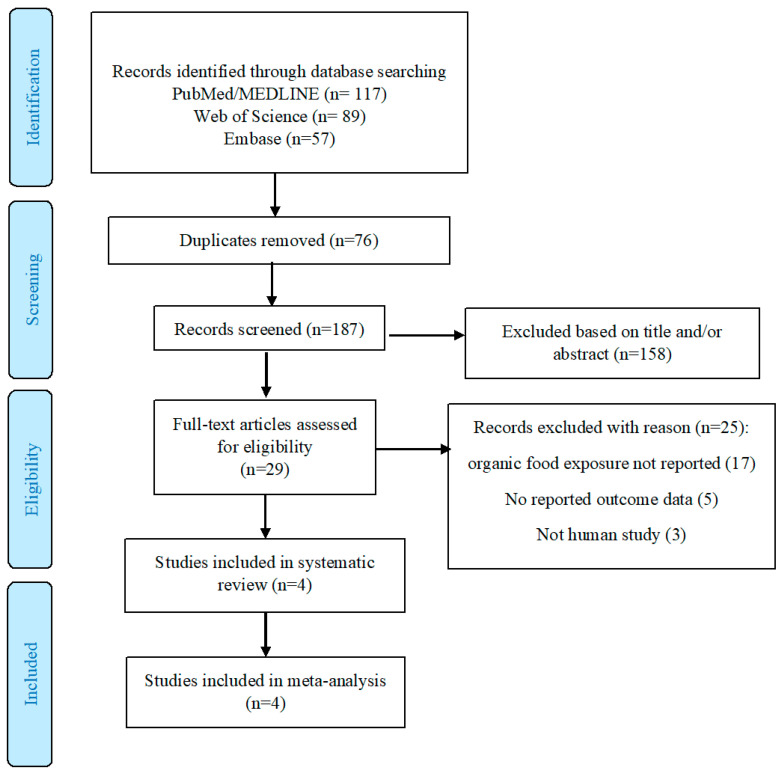
Flow chart of included studies.

The literature review process is demonstrated in a flow chart in Figure 1. The search strategy identified 263 articles: 117 from PubMed/Medline, 89 from Web of Science, and 57 from Embase. Two additional articles were identified by checking the reference lists of included studies. Out of these articles, 76 duplicate records were removed, and 158 articles were excluded following titles and abstract screening. Twenty-five articles were subsequently excluded for various reasons after reviewing the full text. Finally, four studies were included in the systematic review and were eligible for the meta-analysis [18,19,20,21].

Table 1 shows the characteristics of the included studies published between 2010 [21] and 2021 [18]. Two studies were cohort studies [19,20], whereas the remaining two were cross-sectional studies [18,21]. Three were full-length research articles [18,19,21] and one article was published as conference proceedings [20]. Two studies were conducted in France [18,19], while one study each was conducted in five European countries [21] and in the USA [20]. All the studies focused on organic food [19,20,21,22], and one study conducted both children and adults [19]. In total, 104,488 healthy subjects participated in these studies. Regarding the quality assessment of studies, the mean NOS score of studies was 6.8, and the three studies had an overall score of 6 [18,20,21]. More details of the quality assessment are presented in Table 2.

### 4.2. Organic Food Consumption and Risk of Obesity

**Figure 2:**
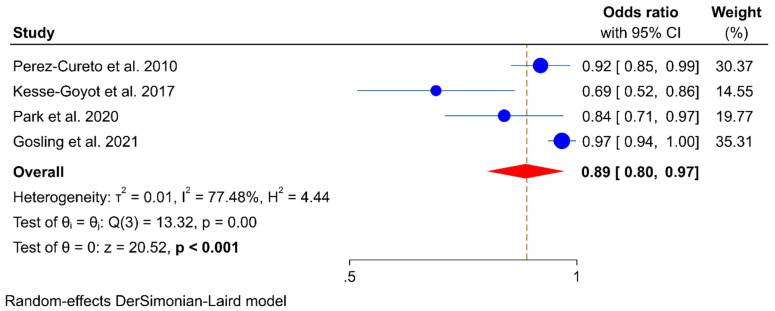
Association between organic food consumption and risk of obesity.

Four studies [18,19,20,21] comprising 104,488 participants with 39,425 adults consuming organic food and 1625 incident cases of obesity were included to evaluate the association between organic food consumption and the risk of obesity. The meta-analysis results showed that organic food consumption was associated with a lower probability of obesity. The pooled OR of obesity for the exposure versus no-exposure groups of organic food was 0.89 (95% CI: 0.80–0.97, *p* < 0.001; *I*^2^ = 77.5%, *P_Q_* = 0.003) (Figure 2).

In subgroup analysis with study population, the pooled ORs in cohort studies and cross-sectional studies [18,21] were 0.78 (95% CI: 0.63–0.92) and 0.95 (95% CI: 0.91–1.00), respectively (Table 3). Subgroup analysis also identified significant heterogeneity between the studies stratified by study design, location, and sample size. The detailed results of the subgroup analysis are shown in Table 3.

### 4.3. Sensitivity Analysis and Risk of Bias

A sensitivity analysis was conducted to investigate the proportion of each study in the overall effect. The findings demonstrated that Pérez-Cueto et al.’s [21] study contributed to between-study heterogeneity in analyzing the association between organic food consumption and the risk of obesity.

## 5. Discussion

This large multinational review of 104,488 participants found that organic food consumption was associated with an 11% reduced probability of obesity. These findings can be explained by several postulates [11,22,23,24]. First, organic foods are considered rich in fruits and vegetables with high amounts of fiber. Second, pesticides, antibiotics, and hormone exposure in conventional food production are much higher, and these chemicals can increase BMI, abdominal fat, and insulin resistance. Third, organic food’s nutrient content and nutritional value could be higher with more vitamins, minerals, antioxidants, or anti-inflammatory ingredients, leading to reduced body weight. Fourth, organic foods are packed with less sugar due to the smaller size of food items than conventional products (e.g., organic fruits of smaller sizes could provide the same amount of nutrients as larger-sized conventionally grown fruits). Finally, it could also be possible that consumers of organic foods could be more health-conscious or have more favorable social determinants of health [22,23,24,25].

Given the billions of dollars being spent on organic food and the growing popularity of such foods, it is imperative to recognize the effects and utility of organic foods. The associations that were observed in this review merit future investigation via prospective and accurate data concerning the nature of foods consumed, which is needed to confirm these findings and assess the long-term effects of organic food consumption on obesity and metabolic disorders. In most of the studies included in this review, the limitations were inherent to study designs (e.g., lack of baseline data, inability to establish cause and effect, recall bias, and issues with reliability and validity of measures). With the multiple geographic and sociocultural influences and individual differences, measuring dietary habits has always remained a challenge. However, to the best of our knowledge, this review is the first of its kind and included participants from various regions; hence, there could be benefits related to body weight from organic food consumption that cannot be ignored.

Data from a descriptive study conducted in Germany demonstrated that most organic food consumers exhibit lower body weight than non-consumers [26]. In addition, a recent Danish study observed that people with generally healthy lifestyles, physical activities, and dietary habits were more likely to eat organic food [27]. Recent studies on organic food consumption have also highlighted that people turned to organic food due to concerns about the potentially harmful effects of pesticide residues on consumer health [28,29,30]. Recent systematic reviews and meta-analyses have indicated that consumers hold positive perceptions largely driven by attributes, such as health benefits, animal welfare, nutritional value, food quality, and food safety [25,31,32]. In addition, several studies published from the NutriNet-Santé cohort have indicated that a higher frequency of organic food consumption was negatively associated with a reduced risk of cancer [33], diabetes [34], metabolic syndrome [35], and obesity [36]. In the present meta-analysis, the association between organic food consumption and obesity remained significant with greater benefits on regular consumption. This variety and extent of evidence also suggest that individuals who consume organic foods, plant-based diets, and whole grains have better cardiometabolic health. Two previous meta-analyses have indicated that switching from conventional to organic dairy and meat products (containing a higher amount of omega-3 fatty acids) benefits the cardiovascular system [37,38]. However, it is still difficult to discern the nutritional significance of organic foods, as most of the included studies are cross-sectional, self-reported organic food frequency, and may have misclassification of organic food consumption. In addition, the evidence on the benefits of organic food remains conflicting given the wide variety of organic foods and the lack of data that has rigorously established that organic foods are more nutritious than non-organic counterparts. For example, organic foods may not necessarily contain fewer calories and may also contribute to high calorie intake (e.g., with newer trends of organic sweets and cookies, etc.). Increasing intake of calorie-rich organic food can pose equal risks of obesity but probably on a reduced scale due to the absence of pesticides or chemicals generally used for non-organic produce. Therefore, the associations observed in this review merit future investigation, with well-designed randomized controlled trials or robust cohort studies.

### Strengths and Limitations

The results of this review should be considered in light of a few potential limitations. First, we included the studies that evaluated organic food consumption through self-reported validated tools (FFQ, 12–24 h recall, and a food-related lifestyle questionnaire) that are susceptible to recall bias. Second, most of the included studies are of moderate quality (NOS score of 6–7), observational, and from Western nations. The participants exhibit specific sociodemographic profiles, and there was significant heterogeneity between the studies. Therefore, caution is needed when generalizing the results. Third, we cannot omit the residual confounding due to the specific profile of high organic food consumers. Fourth, organic food is generally more expensive (specifically in western countries) and it can be reasonably assumed that organic food is mostly consumed by individuals with higher socioeconomic status (SES). These individuals and population groups also have a lower prevalence of obesity; such confounding due to SES factors could limit the validity of our results as there were not enough details across all studies on SES of individuals included for this review. Similarly, organic foods can be of wide varieties (e.g., Non-GMO or antibiotic-free, etc.). It could be possible that our search did not result in studies on such varieties of organic foods or there are no such studies that were conducted. If so, this would bias the findings of our review and limit the validity of our results. Furthermore, obesity is a complex phenomenon with multifactorial origins (e.g., social and cultural preferences for food, market influences, consumer behaviors across regions, etc.). The prevalence of obesity itself varies widely across regions and future studies should account for the wide variation in obesity burden across regions where studies on organic food are conducted. Finally, one of the most critical limitations of this review is that one of the studies included in the review is a conference abstract from the year 2020 and is not yet published as a full-length journal research article [20]. If the review standards for this conference abstract differ from the peer-review standards of other studies included in this review, this could be a threat to the validity of our findings.

Despite these limitations, our review has numerous strengths. First, a comprehensive search was conducted, and selection criteria included all cross-sectional and cohort studies examining the relationship between organic food consumption and obesity. Second, a large sample size, the use of adjusted estimates, stratified analyses, and lack of publication bias enhance statistical power to provide more precise and reliable results. Third, to the best of our knowledge, this is one of the first and largest reviews on the organic food consumption and obesity relationship. Finally, no substantial change in the association between organic food consumption and obesity was found in the sensitivity analysis. Hence, our results remain robust despite these limitations.

## 6. Conclusions

Organic foods have emerged as one of the most popular lifestyle trends over the past few decades. Global sales and consumption indicate further exponential growth of organic food consumption, with a plethora of media reports and lifestyle publications highlighting the benefits of organic foods. Despite the popularity of organic foods, there was a lack of robust evidence on the relationship between organic food consumption and obesity. Our rigorous review of existing evidence and meta-analysis demonstrates a modest reduction in the risk of obesity (11%) associated with organic food consumption. Dietary behaviors and patterns are difficult to measure and quantify, but our review suggests that the impact of organic food consumption on the risk of obesity warrants further investigation through long-term controlled studies. In the global urgency to combat obesity, our study offers promising results for an avenue of intervention and public health education.

## Figures and Tables

**Table 1:** Characteristics of included studies (*N* = 140,067).

Author	Year	Study Design	Country	Study Population	Participants	Sample Size	Method of Assessment	Outcomes	Adjusted	Quality of Studies
Gosling et al. [18]	2021	Cross-sectional	France	INCA3	Children/adult	3896	food propensity questionnaire and 24-h dietary recall	Adult OR: 0.97 Children OR: 0.95	demographics (age and sex), SES (family income and education level), nutritional covariates (MD, adherence, food processing, regime, dietary supplements, and energy intake), and physical activity (physical activity and sedentary lifestyle).	6
Kesse-Goyot et al. [19]	2017	Cohort	France	NutriNet-Santé	Adult	62,224	Organic score/24 h recall	OR: 0.69 (95% CI: 0.58–0.82)	age and sex, occupation, marital status, education, monthly income per unit, dietary supplement use, mPNNS-GS, dietary pattern scores, energy intakes, physical activity and tobacco status, and history of chronic diseases	9
Park et al. [20]	2020	Retrospective Cohort	USA	Sister study	Adult	37,706	organic diet score/12 months recall	RR: 0.84 (95% CI: 0.74–0.96)	socioeconomic, demographic, and lifestyle factors, including physical activity and other dietary measures	6
Perez-Cureto et al. [22]	2010	Cross-sectional	Belgium, Denmark, Germany, Greece, and Poland	Five EU countries (EU FP6)	Adult	2437	Food-related lifestyle questionnaire	OR: 0.92 (95% CI: 0.86–0.98)	age, gender, educational achievement, locality of residence, financial status, and marital status	6

**Table 2:** Assessment of the quality of the eligible studies based on NOS.

Cohort Studies	Selection ^1–4^				Comparability ^5^	Exposure ^6–8^			Total
Kesse-Guyot et al. [19]	1	1	1	1	2	1	1	1	9
Park et al. [21]	1	1	1	0	2	1	0	1	6
**Cross-Sectional Studies**	**Selection ^9–12^**				**Comparability ^5^**	**Outcome ^13,14^**			
Gosling et al. [18]	1	0	0	1	2	1	1		6
Perez-Cueto et al. [22]	1	0	0	1	2	1	1		6

^1^ Representativeness: truly or somewhat representative of the exposed cohort; ^2^ Selection: selection of the non-exposed cohort; ^3^ Ascertainment: assessment by structured interviews or surgical medical records; ^4^ Demonstration: demonstration that outcome of interest was not present at start of study; ^5^ Comparability: study controls for the most important factor or any additional factor; ^6^ Assessment: assessment of outcome; ^7^ Duration: follow-up long enough for outcomes to occur; ^8^ Adequacy: adequacy of follow-up of cohorts; ^9^ Representativeness: truly or somewhat representative of the target population; ^10^ Sample size: justified and satisfactory (including sample size calculation); ^11^ Non-respondents: proportion of target sample recruited attains pre-specified target or basic summary of non-respondent characteristics in sampling frame recorded; ^12^ Ascertainment of the exposure: vaccine records/vaccine registry/clinic registers/hospital records only; ^13^ Assessment: assessment of outcome and their methods validity; ^14^ Statistical test: statistical test used to analyze the data being clearly described, appropriate and measures of association presented including confidence intervals and probability level (*p* value).

**Table 3:** Subgroup analysis.

Subgroup	No. of Studies	Odds Ratio (95% CI)	*p*-Value	*I* ^2^
Study design				
Cross-sectional	2 studies	0.95 (0.91–1.00)	<0.001	38.4
Cohort	2 studies	0.78 (0.63–0.92)	0.007	45.7%
Study location				
Europe	3 studies	0.90 (0.80–0.99)	0.005	81.1%
USA	1 study	0.84 (0.71–0.97)	<0.001	-
Sample size				
<10,000	2 studies	0.95 (0.91–1.00)	0.005	38.4%
>10,000	2 studies	0.78 (0.63–0.92)	0.0001	45.7%

## Data Availability

Data is available from the studies included in the review that have been cited.
